# Comparing multi-image and image augmentation strategies for deep learning-based prostate segmentation

**DOI:** 10.1016/j.phro.2024.100551

**Published:** 2024-02-20

**Authors:** Samuel Fransson

**Affiliations:** a Department of Medical Physics, Uppsala University Hospital, Uppsala, Sweden; b Department of Surgical Sciences, Uppsala University, Uppsala, Sweden

**Keywords:** Segmentation, Deep-learning, Prostate, MR-Linac

## Abstract

During MR-Linac-based adaptive radiotherapy, multiple images are acquired per patient. These can be applied in training deep learning networks to reduce annotation efforts. This study examined the advantage of using multiple versus single images for prostate treatment segmentation. Findings indicate minimal improvement in DICE and Hausdorff 95% metrics with multiple images. Maximum difference was seen for the rectum in the low data regime, training with images from five patients. Utilizing a 2D U-net resulted in DICE values of 0.80/0.83 when including 1/5 images per patient, respectively. Including more patients in training reduced the difference. Standard augmentation methods remained more effective.

## 1 Introduction

In radiotherapy, integrating deep learning (DL) techniques has proven to be a valuable asset in automating contouring tasks, offering reductions in contouring time, and mitigating inter-observer variations [1], [2], [3], [4], [5]. Nevertheless, the successful implementation of such DL networks often hinges on the availability of vast amounts of data and, consequently, a sizable patient cohort for training. Moreover, ground-truth contours typically rely on labor-intensive manual annotation by clinical experts. The landscape of radiotherapy has evolved with the advent of advanced technologies, including Cone Beam Computed Tomography (CBCT)-based imaging and the adoption of Magnetic Resonance Linac systems (MR-Linacs) [6], [7]. Such technologies provide longitudinal data by involving multiple scans for each patient throughout their treatment and may necessitate daily re-contouring to accommodate treatment adaptations.

While some previous studies have leveraged such longitudinal data for patient-specific fine-tuning, resulting in tailored networks for individual patients [8], [9], [10], [11], [12], [13], a deeper examination of the potential advantage of incorporating multiple images per patient into a general auto-contouring network remains somewhat unexplored. Longitudinal images have found their place in some auto-contouring studies for MR-Linacs [14], [15] and other treatments that involve daily imaging and contouring, such as cervical brachytherapy [16], [17], [18]. However, the question of whether multiple images from the same patient can provide sufficient diversity to substantially enhance the performance of an auto-contouring network in comparison to single-image approaches is not investigated. Furthermore, the degree to which this can be compared to established data augmentation techniques is similarly not examined.

In this study, the aim was to investigate to what degree the inclusion of multiple images per patient in the training of a DL auto-contouring network affects their performance and compare this to the utilization of standardized augmentation techniques. Due to the high prevalence of prostate patients treated on our MR-Linac, this work focused on this patient cohort.

## 2 Materials and methods

### 2.1 MR image dataset

A total of 223 T2-weighted MR images were included from 36 prostate patients who received treatment at our Elekta Unity MR-Linac between 2019 and 2023. These patients were treated with a regimen of 6.1 Gy x 7 or 6 fractions, and the use of these images was approved by the Swedish Ethical Review Authority (Approval ID: 2019–03050). Only images from treatment fractions conducted with the Adapt to Shape workflow [6] were included in the study, resulting in an average of 6.2 images per patient, with a range of two to seven images. All MR images were acquired using the same predefined Elekta examcard, applying a T2-weighted 3D turbo spin echo acquisition with 90° flip angle and average repetition/echo times of 1513/263 ms. As online contouring was performed rapidly during treatment, offline re-contouring of the Clinical Target Volume (CTV) (prostate), bladder, and rectum was carried out by a single observer. Given the large anatomical coverage of the images (original image size 300x480x480 voxels with resolution 1x0.86x0.86 mm^3^), occasionally extending well beyond the treatment region, each scan was cropped to a size of 128x256x256 (128 transversal slices) centered around the relevant anatomical structures. This was done primarily to alleviate computational demands and reduce the number of background voxels. In a few instances (12), the number of slices was increased beyond 128 (maximum 148) to achieve broader coverage due to extended structures to a maximum field of view of 148x220x220 mm^3^.

### 2.2 Network design

The U-net architecture [19] was chosen due to its ubiquitous nature and since it has proven start-of-the-art performance for segmentation of medical images. Both a 2D- and 3D-variant were implemented with 30 feature maps in the initial layer, 4/3 (2D/3D) max pooling operations after which the corresponding layer’s number of features doubled, and transposed convolution for upsampling. The activation function for the hidden layers was set to Leaky ReLU (slope 1e-2) and instance normalization [20] was used. The output was obtained following a softmax activation resulting in four output feature maps (background, CTV, bladder, and rectum). The loss function was a combination of equally weighing cross-entropy and DICE as found appropriate for e.g. nnU-net [21].

### 2.3 Training procedure

#### 2.3.1 Multi-image training

**Fig. 1:**
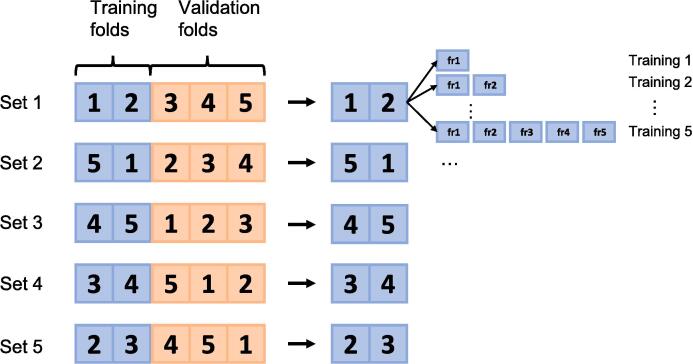
Outline of the training procedure. The training patients were split into five folds (1–5) with all images from five patients in each. For each number of patients included in the training (5/10/15/20), a total of 25 (5x5) models were trained by both varying the patients included in the training data (Set 1–5) and by incrementally adding more fractional images. The example is shown for training with ten patients, where training data is indicated with blue and the validation data with orange, but performed similarly for 5, 15 and 20 patients. (For interpretation of the references to colour in this figure legend, the reader is referred to the web version of this article.)

The MR images were split into training and testing partitions. The training partition encompassed all images from 25 patients, while the testing partition included all images from eleven patients. This division yielded a total of 159 3D images for training and 64 for testing. Within the training data, a further subdivision was performed into five folds, each containing all images from five patients. The training process involved variations in both the number of patients and the number of images per patient. Initially, training was performed with the inclusion of images from five patients and limited to images from their first treatment fraction (resulting in five sets of 3D images). The images from the remaining patients in the training set were reserved for validation. Subsequently, the training dataset was expanded to encompass images from the first two fractions of the same five patients (resulting in ten sets of 3D images). This iterative process continued until five fractional images from each patient were incorporated. This process was performed within the framework of a 5-fold cross-validation setup, repeating these steps with training involving images from five distinct patients at each step. Further training was executed with images from 10, 15, and 20 patients (with the remaining 15, 10, and 5 patients serving as validation, respectively), gradually adding more fractional images per patient. The depicted pattern is illustrated in Fig. 1. Adam optimizer [22] (learning rate 2e-4) was used for training with a batch size of eight transversal images for 2D and two volumes for 3D. Each image was linear intensity normalized to be in the range [0–1]. Training was performed until convergence for each model based on the validation data, without experiencing overfitting for any training. No specific data augmentation other than patch-based training with randomly extracted patches of size 224x224 for 2D and 128x128x128 for 3D was applied in the main experiments listed above. The training was performed with Keras with Tensorflow 2.4 as the backend on an Nvidia RTX 3090 GPU.

#### 2.3.2 Image augmentation training

To see the effect of image augmentation, additional training was performed as above but only with one image per patient. This was to see whether including standard image augmentation techniques had a similar effect to including multiple images per patient. Augmentation included 1) random mirroring in the LR direction with 50 % probability, 2) gaussian noise injection with 50 % probability with mean 0 and standard deviation 0.02, 3) random image scaling, zooming the image with a factor randomly sampled in the interval [1,2] and 4) elastic deformation. The elastic deformation was defined on an 11x11 grid for 2D and 11×11×11 for 3D with randomly sampled deformation vectors between 0 and 4 mm in all directions and applied using the SimpleITK package [23], [24], [25].

### 2.4 Post-processing and evaluation

Each model was applied to every image within the test data partition. During inference, for 2D the entire images (256x256) were processed once, without employing any test-time augmentation or patch-based inference. For 3D, inference was performed with 128x128x128 patches with 64 voxels overlap and a Hann window applied to each patch output to mitigate the decreased accuracy along the borders [21], [26]. The continuous output derived from the softmax activation was subsequently transformed into binary labels by assigning each pixel/voxel to the structure with the highest output probability. Given the requirement for volumetric coherence of all structures in the 3D context, a post-processing step in retaining only the largest coherent volume within each structure was performed. To evaluate the model's performance, the DICE coefficient [27] and the 95 % Hausdorff distance (HD 95 %) were calculated for each obtained structure (A) in comparison to the corresponding reference structure (B). HD 95 % was defined as:

max(HD95%(A,B), HD95%(B,A))

Furthermore, a one-sided paired *t*-test was conducted to assess whether there was a statistically significant improvement when including two or more fractional images in training compared to using only a single image, as well as to determine the potential significance of training with augmentation.

## 3 Results

**Fig. 2:**
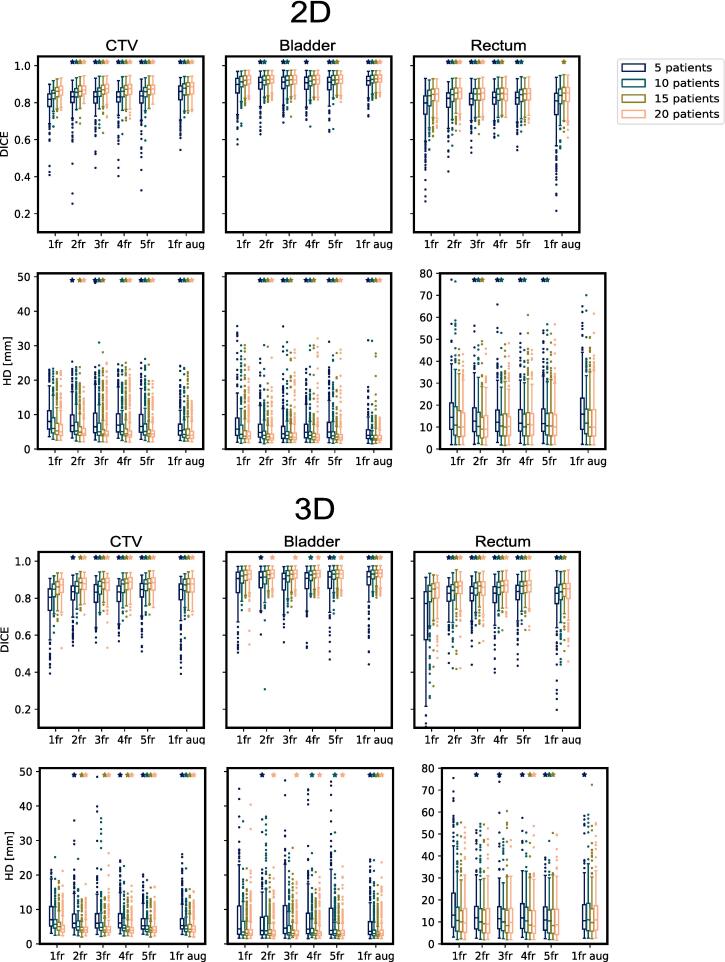
DICE and HD 95 % values of training the networks on different numbers of patients and fractions for both 2D (upper part) and 3D (lower part). The x-axis depicts the number of fractions per patient included in the training, where the rightmost includes augmentation. Bars with different colors slightly separated along the x-axis indicate different numbers of patients included in the training. Outliers are denoted with markers for each box. Stars above each bar indicate whether the results are significantly better (p < 0.05) than results from training with only one fraction (the leftmost group), depicted from a one-sided paired *t*-test. Note the different scale on the y-axis for HD for the rectum. Since the training was performed in a 5-fold cross-validation setting, each bar contains the results from five models.

For the 2D networks, the largest change in median DICE between training with 1/5 fractional images was 0.82/0.84, 0.90/0.91 and 0.80/0.83 for CTV, bladder and rectum, respectively, all found where only five patients were included in the training data. Similarly, for 3D networks, the values were 0.81/0.84, 0.91/0.91 and 0.77/0.84. Comparing training with one fractional image without/with augmentation resulted in 0.82/0.86, 0.90/0.92 and 0.80/0.81 for 2D and 0.81/0.85, 0.91/0.92 and 0.77/0.83 for 3D, also utilizing five patients in the training. Using only one fractional image per patient but utilizing 5/20 patients in training resulted in DICE overlap of 0.82/0.87, 0.90/0.92 and 0.80/0.85 for 2D and 0.81/0.87, 0.91/0.93 and 0.77/0.86 for 3D. In Fig. 2 results in terms of DICE overlap and HD 95 % distance are shown. For clarity, the y-axes are set equal between 2D and 3D, while some datapoints for 3D and rectum appear outside this range. In Fig S1 in supplementary material a range encompassing all results is provided.

## 4 Discussion

In this study, the impact of including multiple images of the same patients within a DL segmentation network for segmentation of the prostate, bladder, and rectum was investigated. It was conducted across various training scenarios, involving different numbers of patients. Additionally, the results were compared with those obtained using standard augmentation techniques. The addition of extra patient scans had only a marginal influence on the performance of the network, as measured by the DICE coefficient and HD 95 %. This observation held true across all experimental settings and for all anatomical structures. Conversely, the utilization of standard augmentation techniques generally led to higher performance improvements in terms of DICE and HD 95 %.

The finding that including more patients in the training cohort had a pronounced impact on the segmentation network's performance is not unexpected [28], as well as the fact that this inclusion had a more profound impact than including multiple images per patient. The inherent anatomical diversity among different patients surpasses the variations seen in the same patient over different days, providing a greater potential for generalization. Similar indications are found in other publications. Chen et al [11] fine-tuned a general network to patient-specific models by progressively adding more fractional images. Fine-tuning with four images vs a single image yielded the same DICE values for CTV and rectum and increased for the bladder with only 0.01. Similarly, Li et al [9] performed fine-tuning for cervical segmentations. For rectum no improvement in DICE was seen utilizing all fractional images for fine-tuning as compared to the average of the first four, while for bladder DICE decreased by 0.02.

Specifically in the context of MR-Linac data, efforts are made to minimize inter-fractional variations in image acquisition settings and patient setup. This minimization facilitates the transfer of structures between images, typically accomplished through deformable image registration, and promotes uniformity in treatment plans across fractions. While some changes in DICE and HD 95 % were statistically significant when incorporating multiple images per patient, as depicted in Fig. 2, the actual effect size was relatively small. This marginal improvement may not always justify the labor-intensive manual annotation required for these additional images and could potentially even be a source of uncertainty if manual delineations between each fraction are performed differently. In such cases, readily available augmentation techniques, as applied in this study, might offer a more practical solution, eliminating the need for further image annotation. For instance, in the case of the prostate, where inter-fractional variation is minimal, augmentation generated greater diversity within the training data, resulting in more substantial performance gains than the mere addition of multiple fractional images. It's worth noting that anatomies characterized by greater inter-fractional variations might potentially benefit more from the inclusion of multiple fractional images. Regarding 2D vs 3D networks, no considerable difference was seen and the same trends applied to both settings. It should be noted that the study’s sample size was relatively limited. Nevertheless, since the impact of including more images per patient was most pronounced when the training dataset comprised very few patients with a diminishing effect with the inclusion of more patients, the overall conclusion appears justified even when considering a larger patient cohort.

In summary, in the context of DL-based auto-contouring on MR-Linac images for prostate treatment, the addition of multiple images from the same patient had only a small effect on segmentation performance, while standard augmentation techniques remain more effective.

## Declaration of Generative AI and AI-assisted technologies in the writing process

During the preparation of this work, the author used ChatGPT from OpenAI in order to improve the readability of the manuscript. After using this service, the author reviewed and edited the content as needed and takes full responsibility for the content of the publication.

## CRediT authorship contribution statement

**Samuel Fransson:** Conceptualization, Methodology, Software, Data curation, Visualization.

## Declaration of competing interest

The author declares that he has no known competing financial interests or personal relationships that could have appeared to influence the work reported in this paper.
