# Supplementary material for: Comparing multi-image and image augmentation strategies for deep learning-based prostate segmentation
Source: Phys Imaging Radiat Oncol. 2024 Feb 20;29:100551. doi: 10.1016/j.phro.2024.100551 (PMC10912785; doi:10.1016/j.phro.2024.100551)
Supplement: Supplementary data 1 [file mmc1.docx]

# Comparing Multi-Image and Image Augmentation Strategies for Deep Learning-Based Prostate Segmentation: Supplementary material

#
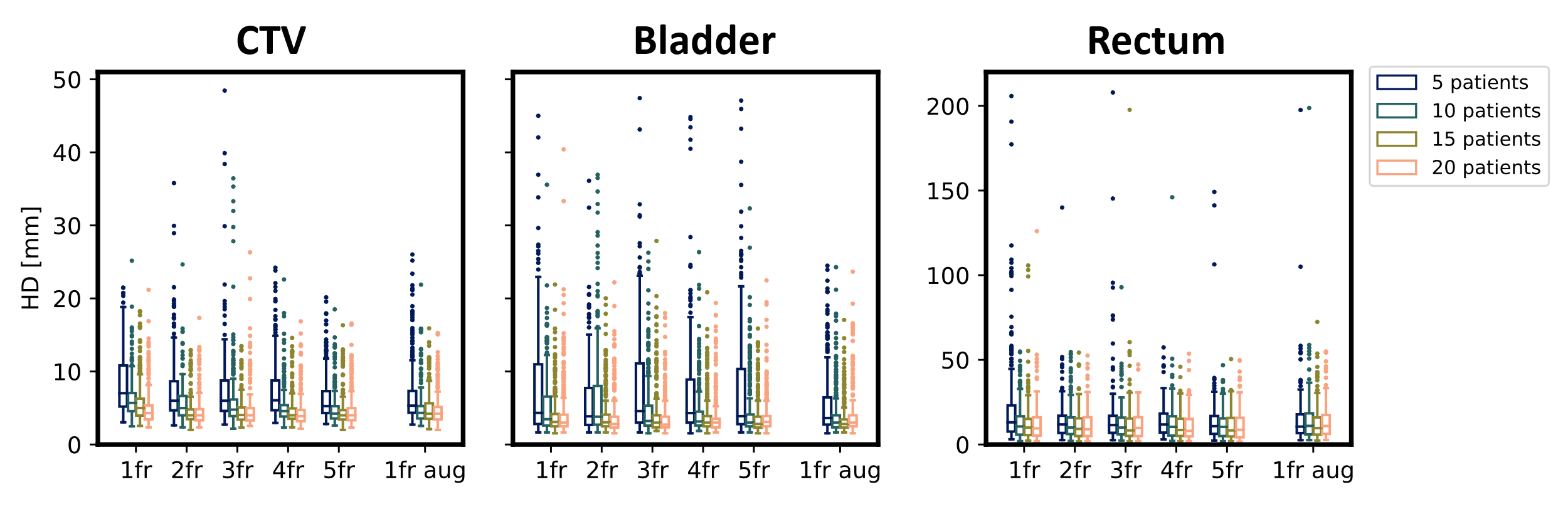


Figure S1. HD 95% values of training the network on different numbers of patients and fractions for 3D network. The x-axis depicts the number of fractions per patient included in the training, where the rightmost includes augmentation. Bars with different colours slightly separated along the x-axis indicate different numbers of patients included in training. Outliers are denoted with markers for each box. Note the different scale on the y-axis for the rectum. Since the training was performed in a 5-fold cross validation setting, each bar contains the results from five models.
